# Innovation in biological microscopy: Current status and future directions

**DOI:** 10.1002/bies.201100168

**Published:** 2012-03-12

**Authors:** Jason R Swedlow

**Affiliations:** Wellcome Trust Centre for Gene Regulation and Expression, College of Life Sciences, University of DundeeDundee, Scotland, UK

**Keywords:** imaging, microscopy

## Abstract

The current revolution in biological microscopy stems from the realisation that advances in optics and computational tools and automation make the modern microscope an instrument that can access all scales relevant to modern biology – from individual molecules all the way to whole tissues and organisms and from single snapshots to time-lapse recordings sampling from milliseconds to days. As these and more new technologies appear, the challenges of delivering them to the community grows as well. I discuss some of these challenges, and the examples where openly shared technology have made an impact on the field.

## Introduction

Biological microscopy has undergone a revolution in the last two decades. Light microscopy in particular is a mainstay of modern molecular, cell and developmental biology laboratories and spans applications in basic research, pre-clinical and recently even clinical domains. Rapid developments at all levels of microscopy experiments – from improvements in sample labelling, contrast, illumination, resolution, signal detection and data processing have all occurred and there is every reason to expect that these advances will simply continue. Applications for these new imaging methodologies are also growing. Combining these technical advances with robotics has resulted in automated microscopes suitable for screening small molecule libraries for drug discovery and recording cell phenotypes that result from systematic gene knockdown by RNA interference (RNAi) or tissue phenotypes caused by diseases. In this overview, I summarise the current state of the field of biological microscopy and highlight some challenges for the future.

## The foundations for modern microscopy: Phase, polarisation and fluorescence

Biological structures are relatively transparent and absorb very little light. Thus one of the biggest challenges in biological microscopy has been the development of methods that generate contrast in biological specimens. Zernike's phase contrast microscopy was the first example where modulation of the illumination pattern generated contrast and revealed biological structure 1. The method discriminates between those rays that have passed through the sample unchanged and those whose path has been changed (i.e. refracted) through interaction with the sample. Refracted rays undergo a phase shift that is used to generate interference. Zernike's phase contrast revealed previously unappreciated detail inside the cell – viewing nuclei and nucleoli in unstained samples and even living samples became routine. A few decades later, Shinya Inoué's application of polarisation microscopy demonstrated the structure and dynamics of spindle fibres in living cells, again taking advantage of the interaction of light with biological structures to generate contrast and reveal the previously unseen 2. Polarisation microscopy had the additional advantage that changes in contrast could be directly related to the underlying biological structure, thus allowing the determination of the structure of macromolecular complexes with the resolution of tens of nanometers 3. Suddenly, the cell was not a simple bag of soluble proteins and nuclei acids, but comprised of defined assemblies of macromolecules and even dynamic polymers. In addition to providing profound insights into biological structure and dynamics, this work demonstrated that an instrument that ‘only’ created a picture of the cell could be used as an exact, quantitative tool that revealed biological structure at the macromolecular scale. For these reasons, Inoué's studies stand as one of the greatest achievements of light microscopy in biology.

Although the principle of fluorescence microscopy was demonstrated early in the 20th century by Heimstädt 4, its utility depended on the development of fluorochromes, secondary labelling and dichromatic mirrors 5. Fluorescence enabled contrast generation through molecular specificity, and opened the door to measurements of molecules within a cellular context in the microscope. The recording of the first three-dimensional (3D) images by optical sectioning fluorescence microscopy by Agard and Sedat 6 provided insight into the higher order structure of cells – chromosomes of the *Drosophila* polytene nucleus had defined, measureable 3D structure and specific interactions between chromatin and the nuclear envelope. However, a major limitation was obvious – the significant blurring of the sample, such that objects near the resolution limit were significantly obscured and severely elongated along the optical axis. Wide-field deconvolution microscopy (WFM) – where a photo-detector like a charge-coupled device (CCD) camera is combined with computational techniques that use a measure of the blurring – the microscope objective point-spread function – to reduce blurring and calculate an image with substantially improved contrast helped reveal even more cellular substructure 7, 8. For thicker objects – large cells, eggs, embryos and tissues, the out-of-focus light dominated any in-focus signal. These samples require a laser scanning confocal microscope (LSCM), where a diffraction-limited spot is scanned across the sample and emitted light is passed through a pinhole that insures out-of-focus light never reaches the detector. These microscopes literally lifted the clouds on many biological samples, enabling visualisation of structure and dynamics by fluorescence microscopy of larger 3D biological systems 9–11.

These early developments demonstrated that microscopy, especially when combined with digital detectors, was a quantitative technique, applicable to a wide range of biological problems, from single molecules all the way to whole organisms. Moreover, they demonstrated that treating digital microscopy as a diffraction-limited process is correct, but substantially underrepresents its potential and application. By introducing specific characteristics to the illumination and/or adding computational processing that considers the point-spread function as a tool, the invisible become easily seen and quantifiable. This is why light microscopy has become such an important part of modern biology.

## Modern methods of microscopy

Since the arrival of the first LSCM and digital WFM systems 8–10, the pace of development and innovation in biological microscopy has only accelerated. The appearance of fluorescent proteins, substantial improvement in detector performance, new illumination modalities that again reveal the invisible or unresolvable have all appeared.

This issue of BioEssays contains reviews of many of these new methods for imaging cells and tissues, including laser scanning, multi-photon, light sheet and super-resolution techniques all of which are now in routine use in research laboratories. Super-resolution techniques are now revealing sub-cellular structures on resolutions that can approach the macromolecular scales and resolving single molecules in cells 12. Light sheet microscopy is now probing the internal architecture of very large embryos and tissues while for the first time providing isotropic and near diffraction-limited resolution in mm-sized samples 13. The latest twist in the application of light sheet microscopy, Bessel beam-based imaging especially in two-photon illumination, promises another significant advance in this field 14. The last few years have seen the rapid development of new spectroscopic techniques that address the underlying dynamics of molecules in living systems. Fluorescence correlation spectroscopy (FCS) and fluorescence cross-correlation spectroscopy (FCCS) are now increasingly used to reveal the size and dynamics of macromolecules in living cells and tissues 15. A technique that sits at the interface between imaging and spectroscopy is quantitative fluorescent speckle microscopy (qFSM) where polymers are labelled sparsely with single molecules and imaging of these and their dynamics, images of their sites and their positions are obtained 16. The images themselves simply show blinking dots and are only converted to real insight with quantitative analysis by image processing. FCS, FCCS and qFSM allow conventional diffraction-limited imaging to reveal the dynamics of molecules at the nanometre scale and thus are critical tools for the biological imaging toolbox.

**Figure 1:**
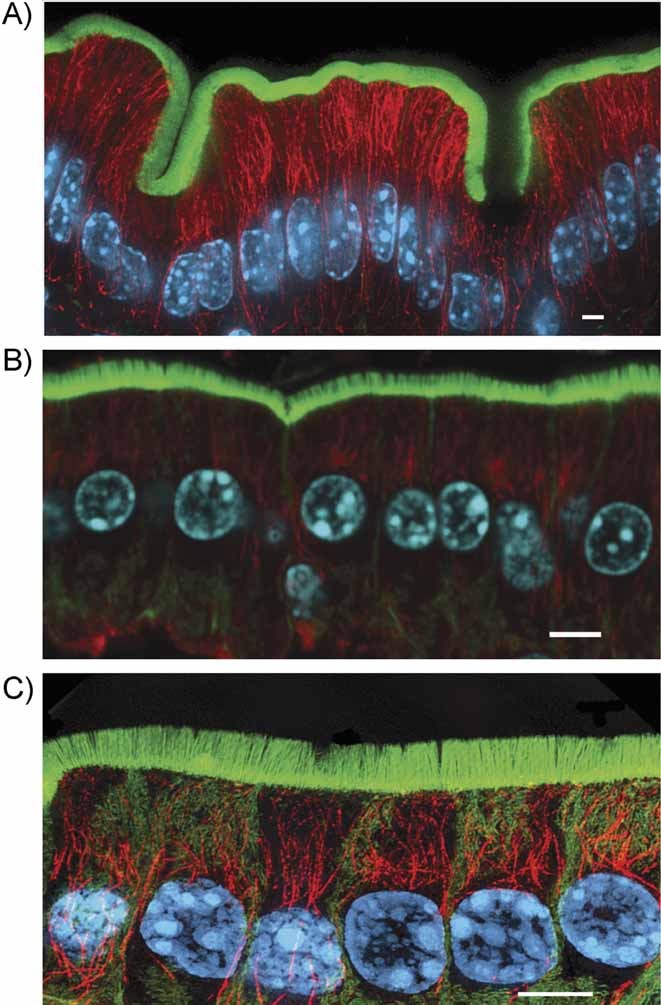
Imaging by WFM, LSCM and 3DSIM. 10 µm section of mouse small intestine, fixed in formaldehyde, cryosectioned, stained with DAPI (blue), anti-tubulin (red) and phalloidin (green) and mounted in glycerol stained with DAPI. **A:** WFM recorded on a Leica fluorescence microscope with a Hamamatsu Orca CCD camera. Scale bar, 10 µm. **B:** LSCM on a Zeiss 710 microscope. Scale bar, 5 µm. **C:** 3DSIM recorded on an OMX microscope. Scale bar, 5 µm. Images courtesy of Paul Appleton and Emma King.

This explosion of parallel techniques recalls the development of LSCM and WFM in the late 80s and early 90s. Initially these approaches were seen to be competitive. Each approach was ultimately demonstrated to have its utility and value on samples where it clearly excelled and was the appropriate tool 17, 18. Other specimens were completely inappropriate and led to unsatisfactory results. Figure 1 shows an example of these differences, using a fluorescently stained section of mouse intestine. WFM properly reveals the structures in the sample, but suffers from out-of-focus light that partially obscures the detail (Fig. 1A). LSCM removes this out-of-focus haze and shows more detail (Fig. 1B). The same sample imaged using 3D structured illumination shows much more detail, even revealing the actin fibrils in the intestinal brush border (Fig. 1C). Similarly, all the new imaging methods should be seen as parts of a set of complementary, expanding tools for probing the structure, dynamics and molecular function of biological systems. In almost all cases, a combination of these tools will be required to fully probe the inner workings of biological cells and tissues.

## New applications for microscopy

As new imaging methodologies come on line, opportunities to apply them to applications beyond their original intent often appear. In the following sections, I explore two especially important applications for many different imaging modalities, screening and intravital imaging.

## Screening by imaging

High-throughput (HT) assays, based on multi-well plates traditionally have been used to perform systematic measurements of in vitro binding, enzymatic activity or gene expression. Cell-based HT assays generate a single value that represents the sum of the signal from all cells in a well. By combining digital microscopy – wide-field, confocal, multi-photon, FCS, etc. – with a motorised stage, image data can be recorded from multi-well plates or from large format slides that have been printed with arrays with transfectable plasmids or siRNAs 19–21. This approach, referred to as high-content screening (HCS) is now routinely used for systematic perturbation or expression studies. Using imaging, the phenotype(s) of each cell in a well are recorded, producing much richer and more detailed datasets. More sophisticated analyses are then possible, where sets of features can be compared to identify phenotypic classes using machine learning techniques 22–24. By staining cells with appropriate reporters, the signatures of specific pathways in individual cells can be measured. This approach is especially important for examining cellular phenotypes after systematic knockdowns of either parts or whole genomes and/or treatment of libraries of small molecules.

High-content screening data are usually recorded at moderate resolution, often in fixed cells. Some systems use confocal optics to improve contrast, especially where high-resolution imaging is required. HCS systems have been adapted with environmental chambers and custom software to record time-lapse images progressing through the cell cycle 20, 25. An FCS-based screen of a library of all open reading frames fused to GFP fusions in *Saccharomyces cerevisiae* has been reported 26 and HCS genome-wide and small molecule screens of animals has been successfully achieved. For example, microchambers for holding *C. elegans* in a defined position for imaging and laser microsurgery have been developed and used for screens of factors involved in neuronal regeneration 27.

The data analysis challenges in HCS are especially acute given the large number of images and the complexity of derived analytic data. The use of multi-parameter analysis, where many separate features are combined to define a specific cellular phenotype using an assembly of features (often called a ‘feature vector’) helps identify unique phenotypes and cluster genes with respect to cellular processes they are involved in 23, 24. Open-source software tools are now available for performing this feature calculation and clustering 28, 29.

Use of RNAi libraries for genome-wide screens is complicated by the presence of RNAi sequences that either do not result in efficient depletion, have off-target effects, or that cannot be validated with siRNAs targeting different sequences of the same gene. A common problem in many of the early generations of siRNA libraries was unintended complementarity with 3′-UTR 30, 31. It is certainly important to minimise these effects using appropriate synthetic chemistry and strategies for siRNA design. Regardless, it is probably unlikely that a single siRNA-based screen can reveal a complete set of phenotypes or even comprehensive assignment of genes to pathways and networks. As in classical genetics, these large-scale HCS experiments are best considered primary screens that help in the discovery process. They are rarely if ever saturating, and the false negative rate is significant, so they are tools for generation of leads meriting further study 25. Following up large numbers of potential hits by higher resolution imaging is certainly a sensible approach, however, this still requires automation as the number of hits is often quite large, and manually collecting high-resolution imaging data can be quite laborious and error-prone. Recently, Micropilot, a software tool that monitors image data acquisition from commercial-microscope platforms and identifies events or phenotypes using machine learning techniques has been described 32. This approach should make hit characterisation and follow-up from screening much more tractable.

## Intra-vital imaging

**Figure 2:**
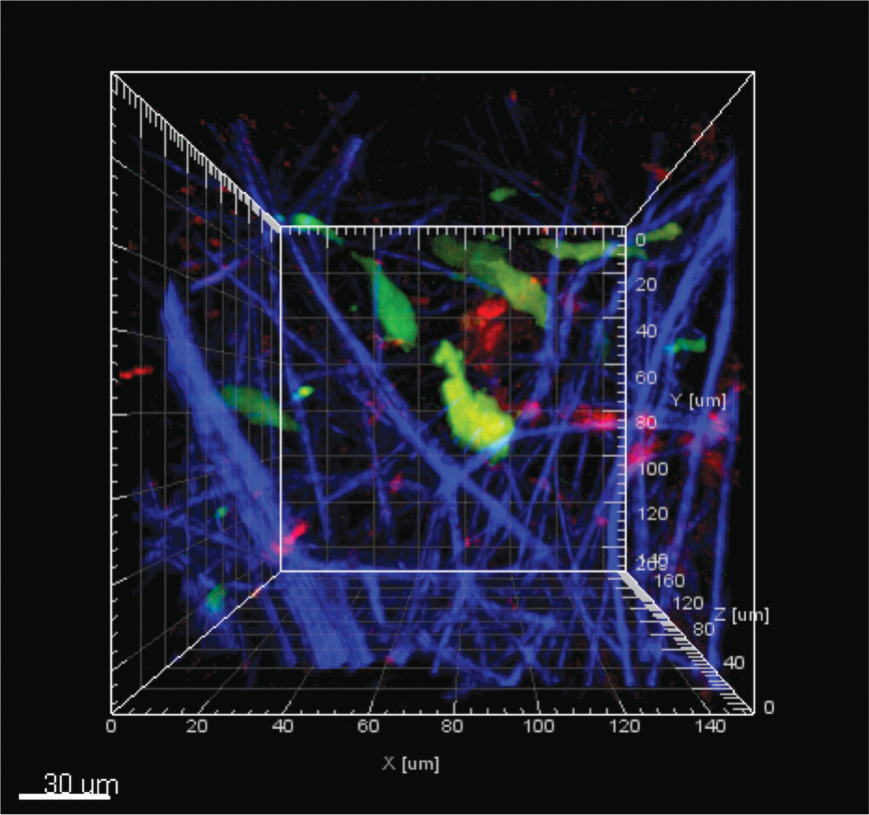
Volume rendered view of three-dimensional data stack of mouse xenograft showing invading p53−/− cells expressing GFP (green), collagen in the extracellular matrix, detected by second harmonic generation (blue), and the vasculature detected by Qdot 655 injected into the tail vein (red). Image recorded using a two-photon microscope. Image courtesy of Max Nobis, Ewan McGhee and Kurt Anderson.

As important as the study of fundamental cell biology is, viewing cells in the context of a whole organism is probably the most definitive way to understand the function(s) and physiology of cells and the pathophysiology of disease. The development of the multi-photon microscope was one of the most important enabling advances in the intravital imaging field, providing subcellular resolution in tissues and in whole organisms 33. The method combines the relatively low absorption of photons in the infrared with the specificity of multi-photon excitation – fluorescence excitation only occurs near the point of focus of the excitation light, since this is the only place where the flux of photons is sufficient to achieve near-simultaneous arrival of two photons at individual fluorophores. Since the excitation volume is restricted to a diffraction-limited point, no confocal aperture is needed – the generation of out-of-focus background is quite limited – and all the excited photons can be collected and measured. The result is good sensitivity, relatively low absorption and 3D imaging at least 500 µm into a tissue (Fig. 2). Realistically, reaching depths beyond 1 mm is usually quite difficult as scattering, aberrations and absorption of the limited fluorescence emission combine to make the resulting fluorescence signal obscured by background. Regardless, the multi-photon microscope has revolutionised many fields, and especially neuroscience, and revealed the structure and dynamics of individual synapses in the living mammalian brain.

For deeper imaging the combination of microscopy and endoscopy, or the so-called microendoscopy, provides access to sub-cellular resolution within the context of living tissues. A number of laboratories have taken this technology forward and demonstrated the use of gradient refractive index lenses attached to the ends of thin optical fibres that can be threaded into living tissues 34–36. The fibre is then connected to an external laser scanning confocal or multi-photon microscope. Most recently a very small 1.9 g microscope has been mounted on the head of an awake behaving mouse, allowing imaging of the blood flow and calcium transients in the brain during normal behaviour 37. This approach promises to reveal the cellular networks that control real behaviour in animals.

## Measuring performance of modern microscopes

Many of the most important discoveries in modern biology would be impossible without the recent developments in microscopy described in this issue. With the proliferation of these new techniques, there is a need to evaluate the performance of separate implementations of the same approach, and measure the differences between different approaches. For example, what are the definitions of sensitivity, resolution and acceptable noise limits in LSCM, and what are the methods for measuring them? An early comparison of LSCM and WFM found substantial differences in the performance of these systems – noise generated by the specific implementations of commercial platforms limited the performance of these systems in ways that were not predicted by a simple consideration of the illumination, detector or light path 17. Subsequent comparison of different realisations of the same imaging method revealed differences in the performance of specific imaging platforms for different applications, and in some cases differences between different realisations of the same method for the same application 38–40. Thus the differences in how different platforms perform define how they can be used, and ultimately the discoveries that can be made with them.

The comparisons of imaging methods that have been published so far were driven largely by frustration – instruments in this and other authors' laboratories did not perform as expected. Unsatisfactory performance immediately translates to incomplete or even unperformed experiments, so there is a need to characterise and understand these limitations. The most comprehensive studies published to date only covered a few examples of a limited number of different imaging modalities, and a few examples of each 38, 39. Murray et al. 38 focussed on one characterisation – the achieved signal-to-noise ratio for a specific sample generated by a specific dose of illumination – and thus is useful in comparing the utility of different imaging modalities for low light-level imaging (e.g. live cell imaging). Probably the most useful outcome of this particular study is the identification of general differences between imaging modalities and the development of a test specimen for comparing 3D imaging systems. However, many more measurements are needed for a complete understanding of system differences and appropriate usage across many domains, and this so far has not been achieved.

Standardisation is common in commercial fields, where a common definition, interface or set of dimensions means that different manufacturers can build systems against a defined standard. A familiar example is the ANSI standard for the design and layout of 96-, 384- and 1,536-well plates for HCS published in 2006 (ANSI/SBS 1-2004 through ANSI/SBS 4-2004; http://www.slas.org/education/microplate.cfm). With the large number of plate, robot and imaging system manufacturers along with many efforts in academic labs, defining a specification for the positions of wells in these plates helps ensure the utility of a wide range of devices, and it properly focuses the field's attention on technology development and scientific discovery. A key player in this development was the Society for Biomolecular Screening, a body representing academic and commercial scientists as well as commercial providers. While this is an important achievement, it is just one example of the standardisation that could happen across the fields of biological microscopy – for detectors, illumination devices and many other components of imaging systems.

## Access to advances in imaging technology

Each of the methodologies highlighted in this issue involves substantial research and development investment and expertise. In many cases, they are the result of close collaboration between biologists, physicists, engineers, software developers and mathematicians. Rather than being the realisation of a single idea, they reflect the power of collaborative teams that collect and assemble technology from many different fields into new tools for biological discovery. As the number and diversity of new imaging techniques and their potential impact on science grows and diversifies, a serious challenge appears for a scientist, lab or even a department facility that wants to access and use new technology for their own research. New technology is often expensive or at least requires significant expertise to develop and assemble. In most cases, the prototypes built in one scientists' lab are functional, but certainly not yet ready to be stable, robust commercial products. Reproducing the collaboration that developed a new imaging system is often impractical. Moreover, new imaging technology may have only been used and proven in a limited range of samples or problems, and its development has not yet benefitted from exposure to a wider range of applications.

The conventional route – where technology is developed in the academic lab and then ultimately licenced to a commercial provider – has produced a number of turnkey imaging systems that are the foundation of modern cell and developmental biology. The commercial provider plays a key role in the development of the final product as they undertake substantial additional development that converts a prototype to a commercial product. However, as the number and complexity of systems now in development is growing, the prototype-to-product pipeline saturates. An additional problem is that some technology platforms may be very expensive to build and require significant expertise to use, and only have limited number of applications. An expensive product with a limited market may not be an attractive proposition for commercial development, but might still deliver capability that is absolutely critical for the scientific community.

These tensions are at the heart of many ongoing discussions within the imaging community and indeed, across many domains in the life sciences 41. Certainly the great popularity of imaging courses run at centres around the world reflect the interest and demand for transfer for knowledge and expertise. One example of a comprehensive effort to ensure access to advanced, innovative imaging technology (at least for European scientists) is the EuroBioimaging project (http://eurobioimaging.eu). Similar large-scale efforts have been successfully undertaken in Australia (http://ncris.innovation.gov.au). Certainly sharing expertise and development and making imaging technologies available to the scientific community as rapidly as possible is critical if the promise and potential of these new technologies and the value of public and private investment are to be fully realised.

## A common bottleneck and a huge opportunity: The data

Despite the growing diversity of methods of imaging, one thing remains in common between them all – they each generate large, complex datasets. Almost always, many steps of processing and analysis are required to convert the original data into a result that can be understood and ultimately published. Most labs use some combination of commercial-, open-source and custom software tools to open, process, view and analyse their data. Invariably, the specific approach for processing and analysis is determined by the specific requirements of the experiment, and thus are not good candidates for standardisation. However, mechanisms for accessing the data, formats and interfaces for processing software can be standardised and shared between scientists, laboratories and institutions, ensuring that efforts in individual laboratories are focussed on the development of their own scientific tools, and not duplicating efforts by others.

Since 2000, the Open Microscopy Environment Consortium (OME; openmicroscopy.org) has developed software infrastructure – file formats, applications and interfaces to enable interoperability between the myriad data formats and processing tools developed by academic and commercial developers – for analysis, management and sharing of biological light microscopy data. OME delivers software that enables interoperability between data, analytic tools and scientists 42. Interoperability could be achieved by explicit links between, for example an application and a single data format, but this makes a brittle, fixed linkage that is not easily adapted to new types of data or analytic tools. In OME, we build a specification, the OME Data Model (http://ome-xml.org) that expresses the data types relevant to an imaging experiment (‘Objective’, ‘Detector’, ‘Laser’, etc.). With this specification as a foundation, we build and release a data format translation plug-in (‘Bio-Formats’) and a data management platform with an open specified interface (‘OMERO’) 43. These tools help standardise data access interfaces, enabling interoperability while allowing data formats and analytic tools to evolve as necessary.

OME is one of a number of open-source image data projects (see Table 1). These projects cover a wide range of functionality, are heavily used by the scientific community and as they are open, there are a number of examples of linkages between these projects. ImageJ, CellProfiler, Endrov and KNIME use Bio-Formats to open image files, and ImageJ, Cellprofiler and Endrov can all open files stored in OMERO. The value of these linkages is just beginning to be realised. For example, as of this writing, Bio-Formats is installed and used by >35,000 sites worldwide. It is not possible to directly measure the impact of this usage, but it is likely that the integration with other tools gives scientists the facility to perform the analyses demanded by their experiments. Because of tools like Bio-Formats, microscopy data repositories like those shown in Table 2 can access image data written in over 100 different formats submitted by the worldwide scientific community and provide rich, sophisticated applications for viewing, searching, annotating and downloading scientific image data.

**Table 1:** Open-source biological microscopy software projects

Project	Description	URL	References
ImageJ	Open-source image visualisation and analysis	http://rsbweb.nih.gov/ij/	[44, 45]
ImageJ2	Re-architecting of ImageJ	http://imagejdev.org	–
Open Microscopy Environment (OME)	Releases Bio-Formats, a file format translator and OMERO, a data management platform	http://openmicroscopy.org	[43]
CellProfiler	Automates feature calculation and analysis, especially for HCS data	http://cellprofiler.org	[28]
Bisque	Web-accessible open analysis framework and a flexible annotation structure for microscopy data	http://bisque.ece.ucsb.edu/	[46]
BioImageXD	Python-based desktop image processing; incorporates ITK image processing functionaity	http://www.bioimagexd.net/	–
Endrov	Open-source multi-dimensional image visualisation and analysis	http://www.endrov.net/	–
Icy	Open-source multi-dimensional image visualisation and analysis	http://icy.bioimageanalysis.org/	–
KNIME	Workflow tools for building simple or complex data processing pipelines	http://www.knime.org/	–
ITK, VTK	Advanced tools for image analysis and visualisation; very popular in biomedical imaging, but applicable to biological microscopy	http://kitware.com	[47]
micromanager	Open-source microscope control platform	http://valelab.ucsf.edu/∼MM/MMwiki/index.php	–
Micropilot	Enables fine control of automated microscopes allowing automated acquisition of specific types of events or structures during fixed cell or time-lapse imaging	http://www.embl.de/almf/almf_services/hc_screeing/micropilot/index.html	[32]

**Table 2:** Open biological microscopy data repositories of biological microscopy image data that take submissions from the community

Project	Description	URL	References
Cell-Centered Database (CCDB)	Provides a repository for well-defined and -annotated image data	http://ccdb.ucsd.edu/	[48]
JCB DataViewer	Provides a repository for original data associated with publications in the JCB. Based on OMERO	http://jcb-dataviewer.rupress.org	[49]
The ASCB CELL Image Library	Provides annotated collections of images of prokaryotic and eukaryotic cells, based on submissions from the community. Based on OMERO	http://cellimagelibrary.org	–
Edinburgh Mouse Atlas of Gene Expression (EMAGE)	A repository for images of gene expression during mouse development. Includes an application for searching user-defined anatomical domains	http://www.emouseatlas.org/emage	[50]

Through open-source release and collaboration, all of these projects in listed Tables 1 and 2 are aware of one another's goals, and are actively taking advantage of each other's technology. As these linkages grow in the next 1–2 years, there will be substantial benefits to scientists who can use these increasingly stable and sophisticated tools for analysis of their data.

## Summary and conclusion

This issue of BioEssays highlights a number of new and emerging technologies in biological microscopy. Most have already had huge impact in basic cell and developmental biology, and applications in drug screening and intra-vital imaging are now being realised. Increasingly sophisticated tools for modulating the illumination of biological specimens improves resolution and contrast in ways that were unimaginable a few years ago. These advances combined with newly developed computational tools are delivering on the promise of imaging in biological research.

The pace of development in imaging brings new opportunities, but it also challenges our current models for delivering innovation to the community. Access to new imaging modalities by the scientific community is imperative to maintain the pace of scientific discovery and ensure that all possible applications, especially in biomedicine are identified and developed. Perhaps the relative success of open-source imaging software is a model that can be used in the future, where open release is combined with defined, accepted licensing models to ensure access for the community, opportunities for synergy and collaboration and the development of new research and commercial applications that drive scientific discovery.

## Abbreviations

CCD: charge-coupled device
FCS: fluorescence correlation spectroscopy
FCCS: fluorescence cross-correlation spectroscopy
FLIM: fluorescence lifetime imaging microscopy
FRET: Förster resonance energy transfer
HCS: high-content screening
LSCM: laser scanning confocal microscopy
OME: open microscopy environment
qFSM: quantitative fluorescent speckle microscopy
RNAi: RNA interference
WFM: wide-field microscopy
